# Growth and Anatomical Parameters of Adventitious Roots Formed on Mung Bean Hypocotyls Are Correlated with Galactoglucomannan Oligosaccharides Structure

**DOI:** 10.1100/2012/797815

**Published:** 2012-05-22

**Authors:** K. Kollárová, I. Zelko, M. Henselová, P. Capek, D. Lišková

**Affiliations:** ^1^Institute of Chemistry, Slovak Academy of Sciences, Dúbravská cesta 9, 845 38 Bratislava, Slovakia; ^2^Department of Plant Physiology, Faculty of Natural Sciences, Comenius University, Mlynská dolina B-2, 842 15 Bratislava, Slovakia

## Abstract

The effect of galactoglucomannan oligosaccharides (GGMOs) compared with chemically modified oligosaccharides, GGMOs-g (with reduced number of D-galactose side chains) and GGMOs-r (with reduced reducing ends) on mung bean (*Vigna radiata* (L.) Wilczek) adventitious roots formation, elongation, and anatomical structure have been studied. All types of oligosaccharides influenced adventitious root formation in the same way: stimulation in the absence of exogenous auxin and inhibition in the presence of exogenous auxin. Both reactions are probably related with the presence/content of endogenous auxin in plant cuttings. However, the adventitious root length was inhibited by GGMOs both in the absence as well as in the presence of auxin (IBA or NAA), while GGMOs-g inhibition was significantly weaker compared with GGMOs. GGMOs-r were without significant difference on both processes, compared with GGMOs. GGMOs affected not only the adventitious root length but also their anatomy in dependence on the combination with certain type of auxin. The oligosaccharides influenced cortical cells division, which was reflected in the cortex area and in the root diameter. All processes followed were dependent on oligosaccharides chemical structure. The results suggest also that GGM-derived oligosaccharides may play an important role in adventitious roots elongation but not in their formation.

## 1. Introduction

The research in the recent years has focused on ascertainment of various plant growth regulators or some chemical products influence on growth and development of agricultural plants [1, 2]. Mung bean is in tropical countries a common and widely cultivated nutritious legume crop with antioxidant activity [3], seedlings of which have been used as a model to examine adventitious root formation [4, 5]. Adventitious root formation is important for the vegetative propagation of plants and their growth. Various plant growth regulators have been tested for rooting of mung bean hypocotyl cuttings [5–7]. Besides growth regulators, oligosaccharides isolated from plant cell walls are the most important factors acting in plant growth and development [8]. Xylooligosaccharides stimulate, for example, the rooting of birch and black pine shoots [9] and induce callus formation and somatic embryogenesis in explants of common mallow (*Malva silvestris* L.) and cotton [10]. Oligogalacturonides support root elongation growth of lettuce [11] and were shown to promote cytokinin-induced vegetative shoot formation from tobacco leaf explants [12]. Trisaccharide fragment of xyloglucan stimulated callus growth and increased the number of embryos in suspension culture of cotton [13]. Hepta- and octa-saccharide (linear oligomers composed of glucose and mannose) isolated from the water extract of the rhizomes of *Paris polyphylla *var.* yunnanensis* stimulated shoot formation of *P*. *polyphylla *var.* yunnanensis* and root hairs growth of *Panax japonicus *var.* major* [14]. A pentasaccharide synthesized by *Paris polyphylla *var.* yunnanensis* showed a significant stimulus on tobacco seedling growth [15].

Galactoglucomannan oligosaccharides (GGMOs) derived from plant cell walls galactoglucomannan influence growth, developmental processes, and defence reactions in plant cells [16–18]. GGMOs showed inhibition effect on elongation growth of pea and spruce stem segments induced by auxins and gibberellin at very low concentrations [19, 20] and their inhibitory effect depended on their chemical structure [20, 21]. GGMOs also inhibited adventitious root formation and elongation of mung bean hypocotyl cuttings in the presence of auxins [22]. Morphology and anatomy of *in vitro* cultivated *Karwinskia humboldtiana* root culture was examined, and the results have shown a dependency on GGMOs concentration and interaction with certain type of auxin [23]. However, the effect of chemically modified forms of GGMOs on adventitious root formation and elongation in plant cuttings has not been studied yet. Therefore, the aim of our work was to compare the effect of GGMOs and their modified forms GGMOs-r (with reduced reducing ends) and GGMOs-g (with reduced number of D-galactose side chains) alone, or in combination with auxins (IBA or NAA) on mung bean adventitious roots formation, elongation, and their anatomy.

## 2. Materials and Methods

### 2.1. Preparation of Galactoglucomannan Oligosaccharides (GGMOs)

GGMOs with d.p. 4–8 were obtained from spruce galactoglucomannan by partial acid hydrolysis as described previously [24]. Galactoglucomannan consists of a backbone of (1 → 4)-linked *β*-D-mannopyranosyl and *β*-D-glucopyranosyl residues distributed at random, having single stubs of (1 → 6)-linked *α*-D-galactopyranosyl residues attached to both mannosyl and glucosyl residues, with slightly preferred substitution of mannosyl residues. GGMOs consist of galactose (4.5%), glucose (21.1%), and mannose (70.4%). Galactoglucomannan oligomers (d.p. 4–8) were composed of tetramers (46%), pentamers (28%), hexamers (12%), heptamers (9%), and octamers (5%). Their number-average molecular mass (*M*_*n*_) was calculated to be 827.

### 2.2. Preparation of Partly Degalactosylated Galactoglucomannan Oligosaccharides (GGMOs-g)

GGMOs-g, with reduced number of D-galactose units to about 50%, were prepared by treatment of GGMOs with purified *α*-galactosidase (EC 3.2.1.22) from coffee beans (Sigma Aldrich, St. Louis, MO, USA) as described previously [25]. Monosaccharide analysis of GGMOs-g by glucose revealed the presence of galactose (2.4%), glucose (21.6%), and mannose (72.0%) residues. Not complete splitting of side chains (deglycosylation only to 47%) is a phenomenon, which may occur by exoenzymes digestion, in this case by the cleavage of *α*-linked galactose residues with *α*-galactosidase. The most plausible causes for this state are inhibition of the reaction by the end product, or steric properties of the molecule. The structural features of the individual oligomers in this mixture did not change in comparison with GGMOs.

### 2.3. Preparation of Modified Oligosaccharides (GGMOs-r)

Modified oligosaccharides GGMOs-r (with reduced reducing ends) were prepared by method described previously [26]. GGMOs of d.p. 4–8 were dissolved in distilled water and treated with 2 M solution of NaBH_4_. Excess of reagent was destroyed by Dowex (H^+^), filtered, concentrated to dryness, and the boric acid was removed by codistillation with methanol. Modified oligosaccharides were dissolved in distilled water and freeze-dried. The mutual ratio of single oligomers in GGMOs-r was the same as in nonmodified GGMOs.

### 2.4. Plant Material and Growth Conditions

Seeds of mung bean (*Vigna radiata* (L.) Wilczek var. Emmerald) (Breeding Station Co., Horná Streda, Slovakia) were soaked in water for 3 hours and sown on cellulose wadding. The seeds were kept in the thermostat for 72 hours at 27 ± 1°C, 80% relative humidity in the dark. Uniform seedlings with 6-7 cm long hypocotyls were cut 5 cm below the cotyledons and roots were removed. For precise dosing, the bases of hypocotyl cuttings were immersed for 24 h in test solutions according to effective and simple method for promoting adventitious root formation [6, 7]. The following treatments were used: IBA and NAA in 10^−4 ^M concentration either alone or in combination with GGMOs and/or with their modified form (10^−8 ^M). IBA, NAA, and GGMOs in their most effective concentrations tested previously were applied [22]. For control variant, distilled water was used. After the treatment with test solutions, cuttings were grown in the substrate (wet sand + peat in the ratio 3 : 1). This substrate is suitable for easy extraction of roots, which is needed for structural studies. Cultivation conditions were the following: 27 ± 1°C, 60–70% relative humidity, 12 h photoperiod, irradiance of 180 *μ*mol m^−2^ s^−1^, and daily watering to maintain constant water saturation of the substrate at cca 75%. Number and length of adventitious roots and their anatomy were determined after six days of growth.

### 2.5. Microscopy

For light microscopy, root segments (3.5–4 mm from the apex in the case of roots treated in GGMOs/GGMOs-g alone or in combination with IBA, and 1 mm from the apex in the case of roots treated in NAA or GGMOs/GGMOs-g in combination with NAA) were fixed in 5% glutaraldehyde and postfixed in 0.5% osmium tetroxide, both in 0.1% sodium cacodylate buffer (pH 7.2). The samples were dehydrated in ethanol and propylene oxide, embedded in Spurr medium, and cut with glass knives using Tesla BS 490 ultramicrotome. Semithin sections made at the distance 3 mm from the root apex were stained with toluidine blue and basic fuchsin [27]. Microscopic samples were recorded with digital camera Sony Exwave HAD. Adventitious root morphometric parameters were determined by Lucia image analysis system (Lucia 4.8, 1991–2002 Laboratory Imaging, Prague, Czech Republic). Diameter of roots (*μ*m), area of rhizodermis, cortex, endodermis, central cylinder (*μ*m^2^), and number of primary cortical cells were measured on the root cross sections.

### 2.6. Statistical Analysis

The values represent the means of three separate experiments with 15 samples per treatment. The data were evaluated by analysis of variance (ANOVA), and comparisons between the mean values were made by least significant difference (LSD) test at *P* < 0.05, and standard error (SE) was calculated.

## 3. Results and Discussion

### 3.1. Root Formation and Elongation

GGMOs stimulated adventitious roots formation in the absence of auxins, though their effect was weaker compared with IBA and NAA (Table 1). On the contrary, in the presence of exogenous auxin GGMOs inhibited adventitious roots formation. All forms of oligosaccharides influenced adventitious roots formation in the same range, no significant differences were determined. Effect of GGMOs on adventitious root formation was independent on their chemical structure.

**Figure 1:**
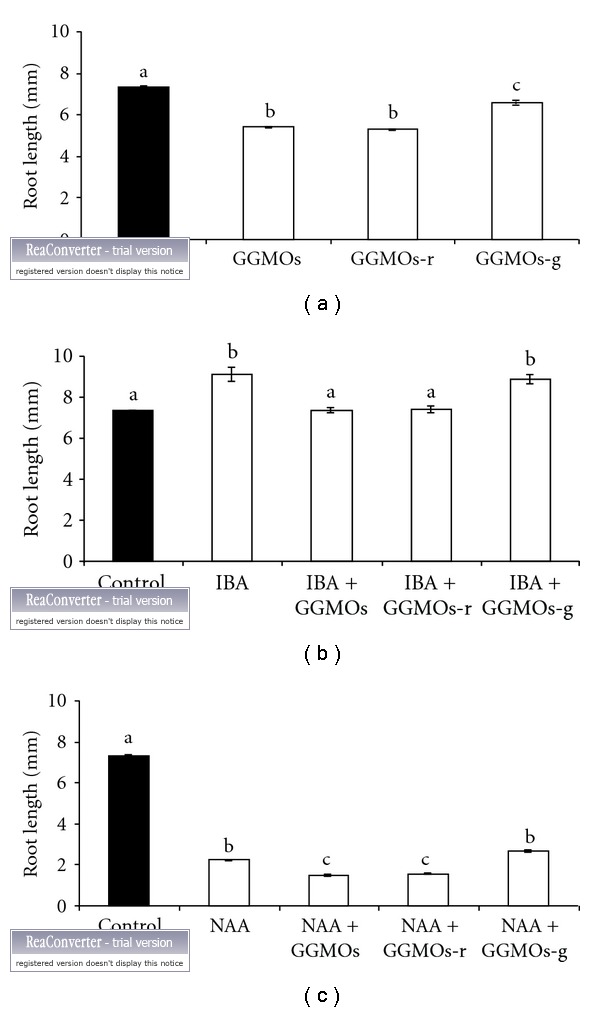
Effect of GGMOs, GGMOs-g, and GGMOs-r alone, and in combination with IBA or NAA on mung bean adventitious root elongation. Control—without any plant growth regulators. Different letters above bars indicate significant differences at *P* < 0.05 according to LSD test.

**Figure 2:**
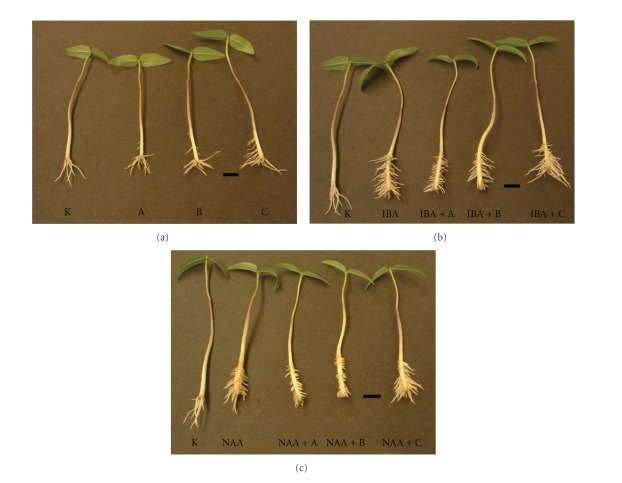
Effect of GGMOs, GGMOs-r, and GGMOs-g alone, and in combination with IBA or NAA on the rooting and adventitious root elongation of mung bean hypocotyl cuttings. K: control, without any plant growth regulators, A: GGMOs, B: GGMOs-r, C: GGMOs-g, bar = 1 cm.

On the other hand, GGMOs and GGMOs-r inhibited root elongation in the absence, as well as in the presence of IBA or NAA, while GGMOs-g inhibition was significantly weaker compared with GGMOs (Figure 1). Moreover, GGMOs-g + IBA and IBA stimulated adventitious root elongation compared with the control (Figures 1 and 2). The impact of GGMOs and their modified forms on adventitious root elongation in the presence of IBA or NAA may be connected with the distinct action of these auxins in the rooting process [28, 29], as well as with their interaction with oligosaccharides used [30]. In addition to this, both reactions (formation and elongation of roots) are probably related with the presence or content of endogenous auxin in such plant cuttings. The reducing ends of GGMOs did not influence their action in root elongation growth similarly as reducing ends of glucan and chitin oligosaccharides did not affect their biological activity [31, 32], while Spiro et al. [33] observed that the modification at the reducing end of oligogalacturonides influenced in different ways their biological activity in morphogenic bioassays. It seems that the inhibitory effect of GGMOs on root elongation could be related to the presence of galactosyl side chains likewise their inhibitory effect in pea stem segments [20]. Similarly, the stimulating or inhibiting effects of oligogalacturonides on root formation in thin-layer explants of buckwheat were dependent on the monosaccharide content [34]. The biological activity of xyloglucan oligosaccharides in plant growth and development was dependent also on their chemical structure [35, 36]. It is evident that the GGMOs chemical structure influences their action in elongation growth of aboveground plant parts [20] and of roots but has no effect on root formation.

### 3.2. Root Anatomy

**Figure 3:**
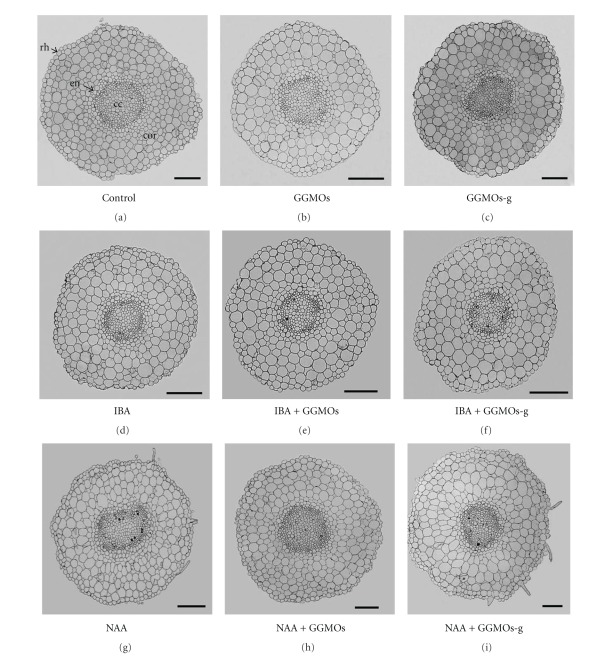
Cross sections of mung bean adventitious roots. Control—without any plant growth regulators, rh—rhizodermis, cor—cortex, en—endodermis, cc—central cylinder, bar = 100 *μ*m.

Differences in structural aspects of adventitious roots were compared from samples cultured in the presence of auxins, GGMOs, GGMOs-g, and under the coaction of auxins with GGMOs or GGMOs-g. The impact of GGMOs-r on adventitious root structure is not shown because GGMOs-r did not influence the root elongation compared with GGMOs. After the GGMOs treatment, it has been ascertained that the diameter of roots, cortex area and central cylinder, and the number of cortical cells decreased in comparison with the control, though in GGMOs-g treated roots these parameters were higher compared to GGMOs treatment (Table 2, Figure 3). From results obtained, it can be supposed that GGMOs inhibit not only adventitious root elongation but also the enlargement of root diameter connected with the inhibition of cortical cells division.

The effect of GGMOs in the presence of both types of auxin on adventitious root anatomy was significantly different in comparison with the previous experiment. GGMOs + IBA increased the diameter of roots, cortex area and central cylinder, and the number of cortical cells in comparison with IBA-treated roots (Table 2, Figure 3). The diameter of roots, cortex area and central cylinder, and the number of cortical cells was significantly lower in the presence of GGMOs-g + IBA compared with GGMOs + IBA. Adventitious roots treated with GGMOs + NAA were larger in diameter, cortex area and central cylinder, and number of cortical cells compared to NAA. Treatment of GGMOs-g + NAA increased the diameter of roots, cortex area, and the number of cortical cells in comparison with GGMOs + NAA. From results obtained, it can be supposed that GGMOs influence cortical cell division likewise in the case of GGMOs action in zinnia xylogenic cultures [37]. The impact of GGMOs on cortical cell division in the presence of different types of auxin is dependent on the chemical structure of oligosaccharides, but probably also on different mechanisms of action of certain type of auxin [38–40].

From our results, it can be concluded that galactose side chains can notably modify the biological activity of GGMOs in elongation of adventitious roots, but not in their formation. The anatomy of adventitious roots affected by GGM-derived oligosaccharides of different chemical structure and combination with certain auxin was then reflected in the root diameter resulting from variations mainly in the cell number and the dimension of cortex area.

## Figures and Tables

**Table 1:** Effect of GGMOs, GGMOs-g, and GGMOs-r alone, and in combination with IBA or NAA on mung bean adventitious root formation. Control—without any plant growth regulators. Means followed by the same letters are not significantly different at *P* < 0.05 according to LSD test.

	Adventitious roots number
Control	10.26 ± 0.04 a
GGMOs	13.82 ± 1.20 b
GGMOs-r	13.93 ± 0.65 b
GGMOs-g	13.41 ± 0.05 b

IBA	55.71 ± 0.96 c
IBA + GGMOs	49.23 ± 0.54 d
IBA + GGMOs-r	46.00 ± 1.80 d
IBA + GGMOs-g	48.13 ± 1.97 d

NAA	50.20 ± 1.37 d
NAA + GGMOs	40.62 ± 2.49 e
NAA + GGMOs-r	40.40 ± 2.21 e
NAA + GGMOs-g	40.31 ± 3.05 e

**Table 2:** Effect of GGMOs, GGMOs-g alone, or in combination with IBA or NAA on the root diameter, area of rhizodermis, cortex, endodermis, central cylinder, and number of cortical cells of mung bean adventitious roots. Particular tissues were measured on transversal root sections. Means in each column followed by the same letters are not significantly different at *P* < 0.05 according to LSD test.

	Root				Area of rhizodermis				Area of cortex				Area of endodermis				Area of central cylinder				Number of			
	diameter (*μ*m)				(1000 *μ*m^2^)				(1000 *μ*m^2^)				(1000 *μ*m^2^)				(1000 *μ*m^2^)				cortical cells			
Control	643.1 ± 7.8 a				23.84 ± 1.06 a				259.51 ± 4.40 a				5.44 ± 0.92 a				25.54 ± 2.66 a				411.5 ± 14.5 a			
GGMOs	518.2 ± 14.2 b				21.57 ± 3.47 a				153.31 ± 3.65 b				4.71 ± 0.52 ab				18.19 ± 0.59 b				244.0 ± 2.3 b			
GGMOs-g	644.4 ± 9.1 a				22.88 ± 2.37 a				249.01 ± 4.11 ac				6.27 ± 0.62 a				23.65 ± 1.15 a				319.3 ± 15.3 c			

IBA	422.4 ± 1.4 c				15.01 ± 0.27 b				106.89 ± 4.20 d				2.91 ± 0.10 b				8.21 ± 0.17 d				186.0 ± 6.0 d			
IBA + GGMOs	469.9 ± 7.7 d				15.40 ± 1.60 b				135.19 ± 3.71 b				3.42 ± 0.10 b				12.18 ±1.14 c				247.3 ± 6.8 b			
IBA + GGMOs-g	426.5 ± 0.9 c				13.73 ± 0.92 b				108.40 ± 1.64 d				3.20 ± 0.08 b				10.95 ± 0.42 ad				197.7 ± 6.3 d			

NAA	596.4 ± 13.2 e				23.48 ± 1.60 a				224.44 ± 7.38 c				8.94 ± 0.25 c				31.87 ± 1.77 e				294.3 ± 5.8 c			
NAA + GGMOs	655.4 ± 20.2 a				28.84 ± 1.17 c				307.59 ± 1.65 e				9.07 ± 1.24 c				37.26 ± 0.80 f				357.0 ± 15.2 e			
NAA + GGMOs-g	761.5 ± 34.4 f				32.09 ± 1.14 c				426.84 ± 2.49 f				8.61 ± 0.77 c				38.89 ± 0.07 f				398.3 ± 22.3 a			

## Abbreviations

d.p.: Degree of polymerisation
GGM: Galactoglucomannan
GGMOs: Galactoglucomannan oligosaccharides
GGMOs-g: GGMOs with reduced number of D-galactose side chains
GGMOs-r: GGMOs with reduced reducing ends
IBA: Indole-3-butyric acid
NAA: 1-naphthaleneacetic acid.
